# Get together to increase awareness in bronchiectasis: a report of the 2^nd^ World Bronchiectasis Conference

**DOI:** 10.1186/s40248-018-0138-3

**Published:** 2018-08-09

**Authors:** Stefano Aliberti, James D. Chalmers

**Affiliations:** 1 Department of Pathophysiology and Transplantation, University of Milan, Internal Medicine Department, Respiratory Unit and Cystic Fibrosis Adult Center, Fondazione IRCCS Ca’ Granda Ospedale Maggiore Policlinico, Via Francesco Sforza 35, 20122 Milan, Italy; 2 Tayside Respiratory Research Group, University of Dundee, Dundee, DD1 9SY UK

Bronchiectasis is a chronic respiratory disease characterized by a permanent dilation of the bronchi associated with cough, daily sputum production and recurrent episodes of respiratory infections [1]. Bronchiectasis pathophysiology starts with a structural airway damage leading to an impaired mucociliary clearance, subsequent chronic bacterial infection and neutrophilic inflammation, which perpetuates this vicious cycle [1]. For a long period of time, bronchiectasis has been recognized as an isolated radiological finding, while now we understand it as chronic and debilitating disease which requires specific management to improve patients’ outcomes.

A renewed interest in bronchiectasis has characterized the field of respiratory medicine over the past few years. When bronchiectasis has been the main theme of scientific sessions at national or international meetings, rooms are packed with colleagues sharing their intellectual interest in this disease. The multidisciplinary approach which characterizes the management of bronchiectasis naturally predisposes physicians to share their experiences and work together to define common pathways to optimize patients’ care [1, 2]. This is something that rarely happens in our field and is the reason why we have seen such a substantial increase in the awareness of bronchiectasis which has, in turn, led to a better knowledge on different aspects of this disease.

Bronchiectasis is no more a neglected disease, nor even less a rare one. Recent data suggest a prevalence up to 566/100,000 subjects in the general population with an increase of approximately 40% in the past decade [3]. Bronchiectasis significantly impacts healthcare systems worldwide with an annual exacerbation frequency up to 3 per patient per year and has a clear attributable mortality [4]. However, real-life data suggest that, to date, quality of bronchiectasis care remains poor [5]. A new and emerging interest has been promoted on this condition as demonstrated by an increase in research activity and the development of international registries over the past few years [6]. New areas of the disease management have been recently considered and different research and clinical gaps have been identified [7].

This special issue of *Multidisciplinary Respiratory Medicine* is following up the largest meeting ever held exclusively focused on bronchiectasis, the 2nd Word Bronchiectasis Conference (WBC, Milan, Italy, July 2017; www.world-bronchiectasis-conference.org). This congress, like the previous one held in Hannover, Germany, in 2016 (world-bronchiectasis-conference.com), represents the clear evidence of the emergence of bronchiectasis as a key clinical entity. Meetings like the WBCs offer the possibility for different healthcare professionals working on bronchiectasis to get together from different continents and actively discuss fundamental aspects of the disease management. These meetings also offer unique opportunities to strengthen collaborations with patients and regulatory agencies, and for young investigators to publish their work with an exceptional international visibility [8]. Important steps towards a better understanding of bronchiectasis have been taken during both WBCs. In Hannover, an international group of investigators developed a consensus definition of exacerbation for clinical trials involving patients with bronchiectasis [9]. In the same line, a discussion started at the 2nd WBC in Milan between bronchiectasis patients and healthcare professionals on the risk of cross-infection which ended up with the first position statement from the European Bronchiectasis Network, EMBARC/ELF patient advisory group and European Reference Network Bronchiectasis Network on this topic [10].

Bronchiectasis is a heterogeneous respiratory disorder and represents the final pathway of several infectious, genetic, immunologic or allergic diseases [11, 12]. An accurate and prompt identification of the underlying cause is a key recommendation of several international guidelines, including rare diseases such as cystic fibrosis (CF) and primary ciliary dyskinesia (PCD) [13, 14]. The diagnosis of PCD and CF has relevant clinical, socioeconomic and psychological implications, which affect patients’ life, including the possibility to have a specific and multidisciplinary team approach in referral centres, as well as a genetic and fertility counselling and special legal aspects in some countries. Both PCD and CF have been hot topics discussed during the 2nd WBC and are arguments of three different papers published in the present issue [15–17]. The papers by Gramegna and Contarini review current literature on PCD and CF and offer interesting insights on why, when and how to investigate both conditions in bronchiectasis, while Robinson and co-workers summarize structural findings of PCD and highlight the radiological differences between PCD and other causes of bronchiectasis [15–17]. An entire session of the 2nd WBC has been dedicated to non-tuberculous mycobacteria (NTM) as one of the most difficult-to-treat pathogens in bronchiectasis [18]. Several open questions exist on NTM from the microbiological diagnosis to the identification of NTM lung disease in the context of a chronic respiratory disease such as bronchiectasis [19]. Real life data coming from international registries are valuable to understand current practices and design translational research [6]. This is the case of the Italian Registry of Pulmonary NTM (IRENE; www.registroirene.it) whose protocol has been reported in the present issue of the journal [20]. New diagnostic techniques have been presented and discussed at the conference such as shotgun metagenomic sequencing which provides an alternative approach that allows the microbial composition of clinical samples to be described in detail, including the prevalence of resistance genes and virulence traits. Rogers and co-workers described a novel, cost-effective strategy for screening patient cohorts for changes in resistance gene prevalence and identified population-level changes in the relative abundance of specific macrolide resistance genes [21]. This is just one of the examples of how new technologies are advancing our understanding of bronchiectasis and how future multi-omics approaches performed across large multicentre studies might help us in interpreting the heterogeneity of this disease [22]. The 2nd WBC has been one of the most productive meeting exclusively focused on bronchiectasis and helped to pave the way towards an increase in bronchiectasis knowledge which will bring us to Washington DC, USA, where the 3rd WBC will be held next July.
